# Tumor growth effects of rapamycin on human biliary tract cancer cells

**DOI:** 10.1186/2047-783X-17-20

**Published:** 2012-06-21

**Authors:** Matthias Heuer, Nici M Dreger, Vito R Cicinnati, Christian Fingas, Benjamin Juntermanns, Andreas Paul, Gernot M Kaiser

**Affiliations:** 1 Department of General, Visceral and Transplantation Surgery, University Hospital of Essen, Essen, Germany; 2 Department of Gastroenterology and Hepatology, University Hospital of Essen, Essen, Germany; 3 Division of Gastroenterology and Hepatology, Mayo Clinic, Rochester, MN, USA; 4 Department of General, Visceral and Transplantation Surgery, University Hospital of Essen, Hufelandstrasse 55, Essen, 45122, Germany

**Keywords:** Anti-tumor effect, Biliary tract carcinoma, Liver transplantation, Rapamycin

## Abstract

**Background:**

Liver transplantation is an important treatment option for patients with liver-originated tumors including biliary tract carcinomas *(*BTCs*).* Post-transplant tumor recurrence remains a limiting factor for long-term survival. The mammalian target of rapamycin-targeting immunosuppressive drug rapamycin could be helpful in lowering BTC recurrence rates. Therein, we investigated the antiproliferative effect of rapamycin on BTC cells and compared it with standard immunosuppressants.

**Methods:**

We investigated two human BTC cell lines. We performed cell cycle and proliferation analyses after treatment with different doses of rapamycin and the standard immunosuppressants, cyclosporine A and tacrolimus.

**Results:**

Rapamycin inhibited the growth of two BTC cell lines *in vitro*. By contrast, an increase in cell growth was observed among the cells treated with the standard immunosuppressants.

**Conclusions:**

These results support the hypothesis that rapamycin inhibits BTC cell proliferation and thus might be the preferred immunosuppressant for patients after a liver transplantation because of BTC.

## Background

Biliary tract carcinoma (BTC) is the second most malignant liver tumor and one of the 10 most frequent gastrointestinal carcinomas worldwide, causing high numbers of fatalities annually [1,2]. Despite all available treatment options, the five-year survival rate of patients with BTC is less than 20% [3,4].

Liver transplantation (LT) is a therapeutic option for treatment of malignant liver tumors, including extrahepatic BTC. It is a treatment modality, besides liver resection, that offers a curative effect [5-9]. However, post-transplant outcome after LT is hampered by recurrence of the primary disease, especially in the case of tumor recurrence [10]. Tumor characteristics in the explanted liver that characterize a ‘high-risk’ pathology, such as a poorly differentiated tumor or vascular invasion, are widely accepted predictors of a poor prognosis. Hence, there is ongoing interest into research on the impact of immunosuppressive drugs upon tumor recurrence. The ideal immunosuppressive agent would simultaneously act as an immunosuppressive agent while exhibiting antitumor properties. In initial studies, rapamycin has been suggested to be a promising immunosuppressant in this regard [11-13]. It appears to be an alternative to the standard immunosuppressive agents, that is, the calcineurin inhibitors cyclosporine A and tacrolimus. In addition to a comparable immunosuppressive effect, rapamycin has also shown to impart an antiproliferative effect *in vitro*[14,15]. As such, it is of particular interest as an immunosuppressant for patients undergoing LT for treatment of liver-originated tumors like BTC.

Rapamycin, a macrocyclic lactone isolated from *Streptomyces hygroscopicus*, has its own unique mechanism of action. By binding to FK-binding protein 12, it inhibits the functioning of a specific cell regulation protein of cell growth, the mammalian target of rapamycin (mTOR), which causes reduced phosphorylation of p70 S6 kinase further down the signal transduction pathway [16-20]. Thus, the drug acts by inhibiting mTOR, which is critical for the coordination of cellular events required for progression from the G_1_ to the S phase of the cell cycle. This appears to be directly related to the observed antiproliferative effect in tumor cells, which is more or less prominent depending on the pathological BTC subtype and the degree of differentiation. The magnitude of this antiproliferative effect appears to vary with different tumor cells and its subsets [8]. Likewise, the correlation between the rapamycin dosages used and the growth-inhibiting effect is not fully understood; especially in the BTC tumor entities.

The aim of this study was to assess the effect of rapamycin on tumor growth of different BTC cell lines in comparison with the standard immunosuppressive drugs. We demonstrate an antiproliferative effect of different doses of rapamycin on BTC cells, taking into account the established concentrations of rapamycin generally used for *in vitro* and *in vivo* studies.

## Methods

### Cell lines and culture modalities

Two human BTC cell lines, EGI-1 and TFK-1, obtained from the German Collection of Microorganisms and Cell Cultures, were used in this study [21]. The cell line EGI-1 was cultured in Dulbecco’s Modified Eagle Medium supplemented with 10% fetal calf serum, l-glutamine, penicillin and streptomycin, while TFK-1 was grown in Roswell Park Memorial Institute medium using the same supplements.

### Drugs

Rapamycin was acquired from Wyeth-Pharma (Muenster, Westphalia, Germany), cyclosporine A and tacrolimus were purchased from LC Laboratories (Woburn, MA, USA). All drugs were exclusively dissolved in dimethyl sulfoxide to create a stock solution. The final concentrations were achieved by diluting the stock solution in culture medium. The most commonly used calcineurin inhibitor doses were selected for the *in vitro* studies according to physiological efficacy and current recommendations.

### Proliferations assays and fluorescence-activated cell sorting analysis

To examine cell count, cell proliferation and DNA synthesis (fluorescence-activated cell sorting analysis, FACS*),* two different standardized staining methods, carboxyfluorescein diacetate succinimidyl ester (CFSE) and 5-bromo-2′-deoxyuridine (BrdU), were performed.

Initially, cells were plated in six-well plates (approximately 10^5^ cells per well in 5 mL culture medium). After 24 hours, cells were treated with vehicle or different concentrations of immunosuppressants (rapamycin: 1, 5, 10, 20 and 50 ng/mL; cyclosporine A: 10 ng/mL; tacrolimus: 25 ng/mL). After 48 hours of incubation, cells were stained according to BrdU- and CFSE-protocols. Subsequently, fixed CFSE-stained cells (after incubation with 2.5 μg/mL RNase and then 2.5 μg/mL propidium iodide solution) as well as 20,000 BrdU-stained cells were analyzed using a FACS scan flow cytometer (Becton-Dickinson, NJ, USA). All FACS data sets were analyzed and calculated with Win MDI Version 2.8 (Josef Trotter, The Scripps Institute, Flow Cytometry Core Facility) and FlowJo Version 7.6.5 (Treestar, http://www.flowjo.com).

### Statistical analyses

Data are expressed as the mean ± standard error of the mean. and represent at least three independent experiments. Following incubation with vehicle or immunosuppressants, FACS analysis was used to determine the division index after a 24-hour period. One-way analysis of variance was used for statistical analyses of the proliferations assays. Differences were considered as significant at levels of *P* < 0.05.

## Results

### Cell proliferation analyses

**Figure 1:**
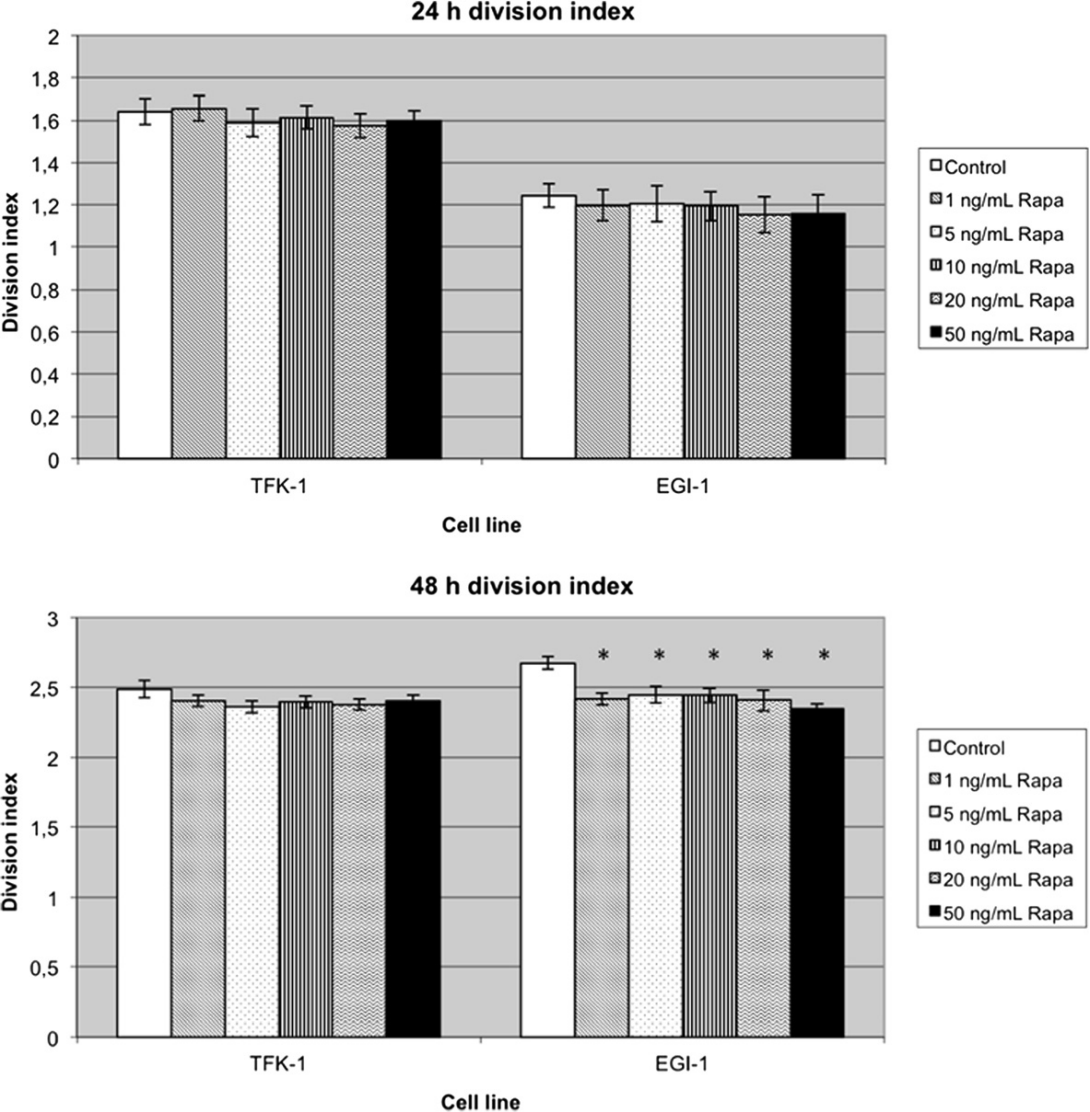
**Proliferation assays showing the division index mean counts of three experiments.** Cells were exposed to rapamycin as described. Rapa = rapamycin. (*P <0.013 versus control).

The cells were treated with immunosuppressants for 24 or 48 hours (Figure 1). The low differentiated BTC cell line EGI-1 showed a homogenous inhibition of the division index in the rapamycin group. The strongest antiproliferative effect was observed at hour 48. Here, cell growth was reduced by 8.5 to 12.4% (*P* = 0.001).

A similar result was observed in the TFK-1 cells treated with rapamycin. In this BTC cell line, the division index found in the control groups (with the exception of 1 ng/mL rapamycin after 24 hours) was always above those of groups treated with rapamycin. As in EGI-1 cells, non-significant differences in growth inhibition between the respective dosages 5, 10, 20 and 50 ng/mL were observed in the TFK-1 cells.

**Figure 2:**
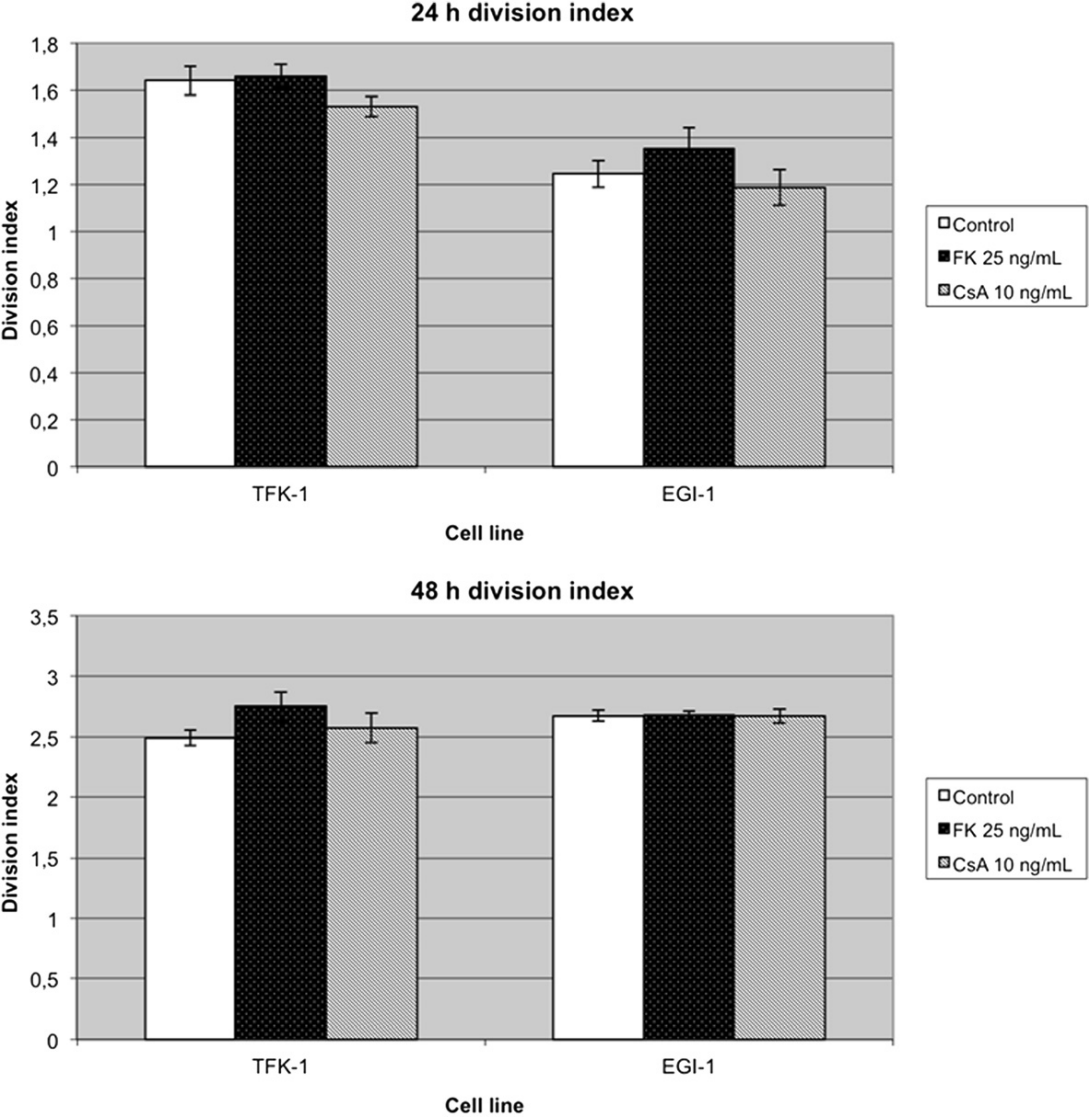
**Proliferation assays showing the division index from mean counts of three experiments.** Cells were treated with tacrolimus or cyclosporine A. FK: tacrolimus; CsA: cyclosporine A.

This examination was completed by repeating the same measurements with the standard immunosuppressants cyclosporine A and tacrolimus. In contrast to the constant antiproliferative effect of rapamycin, both standard immunosuppressants caused a noticeable increase in BTC cell growth (Figure 2) in some cases. Tacrolimus increased cell growth in the low differentiated cell line EGI-1 by 8.6%, while cyclosporine A-treated cells exhibited a growth rate decrease of 4.6% after 24 hours of incubation. In TFK-1 cells, the division index at hour 24 and 48 at 10.4% was noticeably higher in tacrolimus-treated cells compared with the control and cyclosporine A group.

### Cell cycle analyses

These studies were carried out to further analyze the biological basis of the immunosuppressant’s mechanisms of action. Tables 1 and 2 show the various phases of the cell generation cycle after treatment with the immunosuppressants.

**Table 1:** Percentages of EGI-1 in each phase of the cell cycle after 48 hours of exposure to different dosages of rapamycin

**Group**	**Apoptosis**^**a**^			**G0/G1 phase**			**G2 + M phase**			**S phase**		
	**Mean**	**±SE**	***P***^**b**^	**Mean**	**±SE**	***P***^**b**^	**Mean**	**±SE**	***P***^**b**^	**Mean**	**±SE**	***P***^**b**^
control	0.24	0.04		32.56	1.01		22.44	0.52		25.44	0.85	
rapa 1 ng/mL	0.28	0.02	0.33	35.98	1.21	0.55	21.24	0.35	0.085	17.96	0.23	<0.001
rapa 5 ng/mL	0.30	0.02	0.21	38.77	1.28	<0.05	22.21	0.26	0.70	19.97	0.44	<0.001
rapa 10 ng/mL	0.52	0.04	<0.05	37.09	0.50	<0.05	24.45	0.70	<0.05	15.88	0.29	<0.001
rapa 20 ng/mL	0.55	0.04	<0.05	36.75	1.32	<0.05	23.31	0.45	0.23	14.56	0.33	<0.001
rapa 50 ng/mL	0.42	0.05	<0.05	33.16	0.44	0.60	26.05	0.14	<0.05	16.15	0.37	<0.001

Cell cycle analysis of EGI-1 and TFK-1 cell lines 48 hours after treatment with rapamycin at doses of 1, 5, 10 and 20 ng/mL, with 5-bromo-2′-deoxyuridine and 7-aminoactinomycin D staining ^a^Sub G1-region, indicating cells with small DNA fragments, a typical feature of apoptosis; ^b^t-test was performed; while a Levene test was performed in case of unequal variances, *P* <0.05 was considered significant. Rapa: rapamycin; SE: standard error of the mean.

**Table 2:** Percentages of TFK-1 in each phase of the cell cycle after 48 hours of exposure to different dosages of rapamycin

**Group**	**Apoptosis**^**a**^			**G0/G1 phase**			**G2 + M phase**			**S phase**		
	**Mean**	**±SE**	***P***^**b**^	**Mean**	**±SE**	***P***^**b**^	**Mean**	**±SE**	***P***^**b**^	**Mean**	**±SE**	***P***^**b**^
control	0.15	0.03		37.78	1.67		39.00	1.33		8.9	0.34	
rapa 1 ng/mL	0.11	0.02	0.21	51.75	1.07	<0.001	33.72	0.92	<0.05	5.55	0.43	<0.001
rapa 5 ng/mL	0.09	0.01	<0.05	50.74	1.31	<0.001	35.45	0.69	<0.05	4.10	0.16	<0.001
rapa 10 ng/L	0.08	0.01	<0.05	49.53	1.15	<0.001	35.28	0.66	<0.05	4.38	0.30	<0.001
rapa 20 ng/mL	0.15	0.01	0.92	47.26	1.10	<0.001	35.97	0.87	0.09	5.61	0.13	<0.001
rapa 50 ng/mL	0.19	0.02	0.25	49.86	2.17	<0.001	37.67	1.11	0.46	3.21	0.33	<0.001

Cell cycle analysis of EGI-1 and TFK-1 cell lines 48 hours after treatment with rapamycin at doses of 1, 5, 10 and 20 ng/mL, with 5-bromo-2′-deoxyuridine and 7-aminoactinomycin D staining. ^a^Sub G1-region, indicating cells with small DNA fragments, a typical feature of apoptosis; ^b^t-test was performed; while a Levene test was performed in case of unequal variances, *P* <0.05 was considered significant. rapa: rapamycin; SE: standard error of the mean.

Among the rapamycin-treated EGI-1 cells, only a mild increase in the G0/G1 phase was observed as compared with the control group, whereas among TFK-1 cells, the G0/G1 phase was increased up to 37% (51.75% versus 37.78%; *P* ≤0.001). TFK-1 cells treated with tacrolimus and cyclosporine A, respectively, especially showed a decrease in the G1 phase. At the same time, an increase in the S phase and an increase in DNA synthesis were observed in those cells.

Moreover, rapamycin-treated EGI-1 cells displayed a significant increase in apoptosis. Additionally, in both cell types, the synthesis rate (S phase) clearly dropped. The strongest decrease of the synthesis rate (14.56% versus 25.44%; *P* ≤0.001) was observed in the EGI-1 cell line at a dose of 20 ng/mL rapamycin. Conversely, a marked increase in the rate of synthesis and a decrease in the apoptosis rate of TFK-1 cells were observed in tacrolimus-treated cells. An increase of mitotic cells was even more pronounced in cyclosporine A-treated TFK-1 cells.

## Discussion

It is not yet clear how important the role of immunosuppression is in the development of post-transplant BTC recurrences. Some investigators consider the majority of the recurrences to be related to metastatic disease that either was present but unidentifiable prior to transplantation or was caused during the transplant procedure [8]. Other studies indicate that proper immunosuppression management can stabilize the recurrence rate (for example, in hepatocellular carcinoma cell lines) at a satisfactory level [22,23]. Our study clearly demonstrates that rapamycin inhibited the growth of different BTC cell lines *in vitro*.

The post-transplant requirement for immunosuppression appears to facilitate tumor growth. Different studies have shown that the standard immunosuppressive agents cyclosporine A and tacrolimus directly intervene in the tumor cell cycle, inducing an increase in cell synthesis and resulting in the stimulation of tumor cell growth and a subsequent increase in the recurrence rate [24,25]. These calcineurin inhibitors act by reducing interleukin-2 expression, inhibit the early activation of T lymphocytes (that is, the transition from the G_0_ to the G_1_ phase of the cell cycle) and promote tumor cell cycle progression by increasing cdk4 kinase activity [26,27].

Rapamycin acts by inhibiting the mTOR signaling pathway, as described above. This pathway is already known to be upregulated in various carcinoma cell lines, such as lung cancer, renal cancer, ovarian cancer and breast cancer, as well as in rhabdomyosarcoma, B lymphoma and osteosarcoma [28,29]. Therefore, this pathway is of particular interest because of the effective inhibition function of rapamycin on different BTC cell lines. Several studies have indeed shown an activated mTOR pathway in a subset cell line of the liver and an inhibition of proliferation of neoplastic hepatocytes in culture, while molecular biological studies, in which different BTC subtypes are examined with various doses of immunosuppressants, are presently rare [30,31].

The purpose of our study was to examine the inhibiting effect on tumor cell proliferation of differentiated BTC cell lines after treatment with rapamycin and the standard immunosuppressive therapy. Herein, we show that rapamycin inhibited the growth of two BTC cell lines. The reduction of the division index occurred almost independent of the rapamycin doses used; there were marginal differences between clinically used doses versus high doses. Furthermore, rapamycin-treated EGI-1 cells showed a noticeable increase in apoptosis, while the synthesis rate of both BTC cell lines dropped significantly at the same time. By contrast, the calcineurin inhibitors cyclosporine A and tacrolimus induced cell growth in some of the experiments. At all measurement times, immunosuppressant-treated cell lines showed a higher division index than cells in untreated control groups. In cell lines treated with calcineurin inhibitors, the reverse pattern was observed, with these cells exhibiting an increase in rate of synthesis and a decrease in apoptosis rate.

In summary, our observations suggest that rapamycin imparts an antiproliferative effect on BTC cells and therefore might be an advantageous immunosuppressant for patients after LT due to BTC. However, a uniform post-transplantation immunosuppression regimen with rapamycin as a single-agent has not yet been determined. Thus, a combination of known standard immunosuppressive agents with rapamycin appears to be a more suitable option. The specific combination of these immunosuppressive agents would then depend on the type of tumor.

Individual cases have been reported where treatment with rapamycin led to a black box warning. *De novo* immunosuppression with rapamycin after transplantation resulted in disturbed wound healing, artery thrombosis and toxicity. The antiproliferative tumor cell effect of rapamycin demonstrated in this study should still be considered in post-transplantation immunosuppression regimens (LT due to liver-originated tumors like BTC). The first steps in this direction, where a primary therapy of standard immunosuppressive agents is replaced by rapamycin for maintenance immunosuppression, have been undertaken. Rapamycin should not be considered as a chemotherapeutic agent but it might be helpful for the prevention of early tumor recurrences after LT [32].

## Conclusion

Our results support the hypothesis that rapamycin is a more suitable immunosuppressant for patients after LT due to BTC. Future - even *in vivo* - studies will have to investigate the ideal combinations of immunosuppressive agents to provide maximal tumor suppression while ensuring a safe long-term survival free of rejection episodes.

## Competing interests

The authors declare that they have no competing interests.

## Authors’ contributions

MH, carried out the molecular genetic studies, result analyses, and wrote the manuscript. NMD, carried out the molecular genetic studies. VRC carried out the molecular genetic studies, result analyses. CF, cross-read the manuscript. BJ, cross-read the manuscript. AP, cross-read the manuscript. GMK, cross-read the manuscript. All authors read and approved the final manuscript.
